# Cellular Immunotherapy and Locoregional Administration of CAR T-Cells in Malignant Pleural Mesothelioma

**DOI:** 10.3389/fonc.2020.00777

**Published:** 2020-06-03

**Authors:** Robert A. Belderbos, Heleen Vroman, Joachim G. J. V. Aerts

**Affiliations:** ^1^Department of Pulmonary Medicine, Erasmus MC Rotterdam, Rotterdam, Netherlands; ^2^Erasmus MC Cancer Institute, Erasmus MC Rotterdam, Rotterdam, Netherlands

**Keywords:** mesothelioma, cancer vaccines, dendritic cell therapy, CAR-T cell therapy, immunotherapy

## Abstract

Malignant pleural mesothelioma (MPM) is a treatment recalcitrant tumor with a poor overall survival (OS). Current approved treatment consists of first line chemotherapy that only modestly increases OS, illustrating the desperate need for other treatment options in MPM. Unfortunately, clinical studies that investigate the effectivity of checkpoint inhibitor (CI) treatment failed to improve clinical outcome over current applied therapies. In general, MPM is characterized as an immunological cold tumor with low T-cell infiltration, which could explain the disappointing results of clinical trials investigating CI treatment in MPM. Currently, many other therapeutic approaches, such as cellular therapies and cancer vaccines are investigated that could induce a tumor-specific immune response and increase of the number of tumor-infiltrating lymphocytes. In this review we will discuss these novel treatment approaches for MPM.

## Introduction

**Figure 1:**
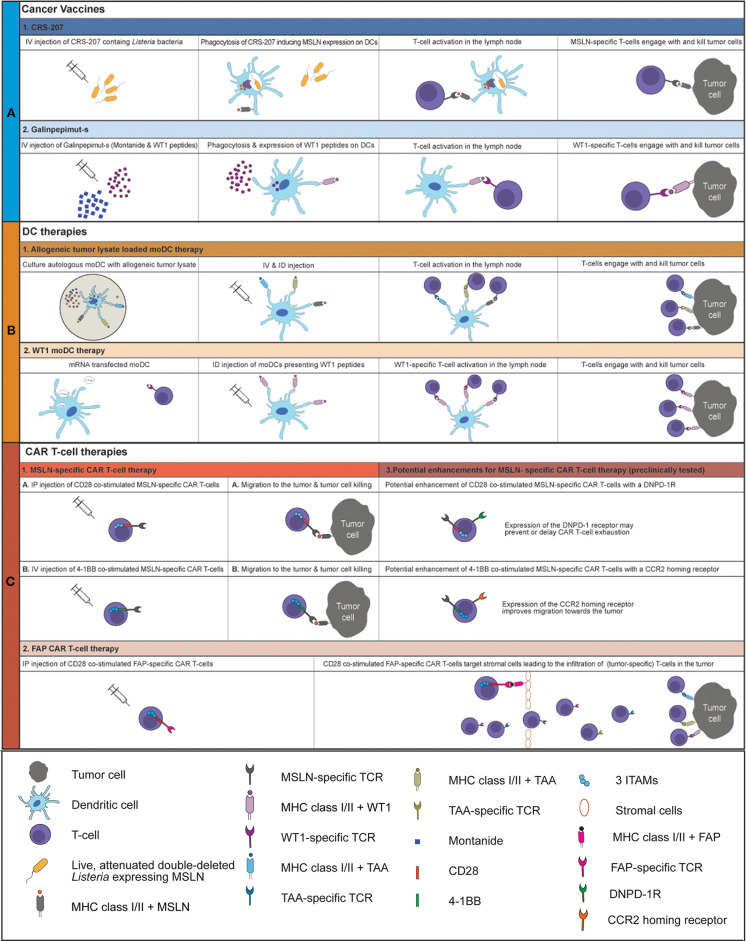
Overview of current clinically tested cancer vaccines and cellular therapies for MPM An overview of the working mechanism of CRS-207 (A1), Galinpepimut-s (A2), allogeneic tumor lysate loaded moDC therapy (B1), WT1 moDC therapy (B2), MSLN-specific CD28 co-stimulated CAR T-cell therapy (C1A), MSLN-specific 4-1BB co-stimulated CAR T-cell therapy (C1B) and FAP CAR T-cell therapy (C2). The potential enhancements of MSLN-specific CAR T-cell therapy are displayed in C3. IV, intravenous; ID, intradermal; IP, intrapleural; MSLN, mesothelin; moDC, monocyte-derived dendritic cell; MHC, major histocompatibility complex; TCR, T-cell receptor; WT1, Wilms Tumor 1 protein; TAA, tumor-associated antigen; ITAM, Immunoreceptor tyrosine-based activation motif; DNPD-1R, dominant negative PD1 receptor; CCR2, CC chemokine receptor 2; FAP, fibroblast activation protein.

Malignant pleural mesothelioma (MPM) is a lethal cancer with limited treatment options (1–3). Current first-line treatment, consisting of platinum/antifolate combination therapy, leads to a median overall survival (OS) of 9–2 months (4). The addition of Bevacizumab to first-line treatment increased OS by 2.7 months and is now the accepted standard therapy in France (5, 6). Since then, no new treatments that could improve the outcome for MPM were reported. Immunotherapies, aiming at the activation of the immune system by blocking inhibitory checkpoint receptors, called checkpoint inhibitor (CI) treatment have drastically improved OS for non-small cell lung cancer and melanoma patients (7). So far, CI treatment has been promising for a small group of MPM patients in phase I/II trials, with response rates between 9 and 29% (8–17). However, unfortunately the DETERMINE phase IIb trial failed to show superiority of anti-cytotoxic T-lymphocyte-associated protein 4 (CTLA-4) (Tremelimumab) over placebo in a second or third-line setting for MPM (18). Moreover, in the PROMISE-meso trial, blockade of programmed cell death protein 1 (PD1) failed to prolong progression free survival (PFS) or OS compared to second-line chemotherapy (gemcitabine/vinerolbine) treatment (19). Combination treatment of monoclonal antibodies (mAbs) targeting PD1 or PD1 ligand (PD-L1) with anti-CTLA4 mAb seems to be more effective than CI monotherapy in MPM (10, 20, 21). The results of the ongoing Checkmate 753 phase III trial are awaited (NCT02899299), where Nivolumab (PD1 blockade) and Ipilimumab (CTLA-4 blockade) are combined as first line therapy in unresectable MPM and compared to first-line chemotherapy consisting of pemetrexed and cisplatin or carboplatin (22). As CI treatment, especially anti-PD(L)1 mAb, reinvigorates T-cells, the low number of tumor-infiltrating T-cells (TILs) in MPM might explain the relatively low response rates found in clinical trials investigating anti-PD1/PD-L1 treatment (23). Tumors with high numbers of TILs respond better to CIs (24). In MPM, dendritic cells (DCs) are reduced in both their numbers and their functionality, which could explain the low numbers of TILs (25). Induction of tumor-specific T-cells that infiltrate tumor and kill tumor cells upon antigen recognition by secretion of perforins, granzymes and death ligands, such as Fas and TRAIL could improve clinical outcomes (26, 27). Cancer vaccines and DC-therapy can induce activation and proliferation of tumor specific T-cells. Additionally, chimeric antigen receptor (CAR) T-cells, specific for a tumor antigen, can be used to target specific tumor antigens directly. Recent developments in therapies initiating a tumor directed immune response, such as cancer vaccines, DC-therapy and CAR T-cell therapy in a clinical setting in MPM will be discussed in this review (Figure 1).

## Cancer Vaccines

Cancer vaccines can be made of tumor lysate, single or multiple peptides, viruses, or attenuated bacteria. The purpose of vaccinating cancer patients is to elicit a tumor-specific type 1-polarized T-cell response, leading to clinical benefit for the patient. Immunostimulatory adjuvants, such as granulocyte-macrophage colony-stimulating factor (GM-CSF) and toll-like receptor (TLR) ligands are often combined with cancer vaccines, to attract and activate antigen presenting cells (APC) that will take up the cancer vaccines (28). Certain adjuvants, such as Montanide, protect the peptides in the cancer vaccine and create a depot for slow antigen release that attracts lymphocytes and DCs, therefore called depot adjuvants (29). For MPM, Wilms Tumor 1 (WT-1) peptide-based vaccine, Galinpepimut-S and CRS-207 are the most thoroughly evaluated cancer vaccines and will be discussed in more detail.

## WT-1 Cancer Vaccines

WT-1 is a protein expressed on almost all (97%) MPM cells with a variable distribution and intensity and serves as an immunohistochemical marker for MPM diagnosis, making WT-1 an appropriate target for immunotherapy (30). The cancer vaccine, Galinpepimut-S consist of four WT-1 peptides of different lengths that can be presented in both MHC class I and II molecules, permitting the activation of both CD4^+^ and CD8^+^ T-cells (31). Treatment with Galinpepimut-S was investigated in a randomized phase II study in MPM patients with positive (> 10%) WT-1 expression. Herein, Galinpepimut-S was administrated with adjuvants (GM-CSF and Montanide) and compared to placebo, in which only the adjuvants were administered. Unfortunately, the study was closed after inclusion of 41 patients due to futility of the placebo treatment and a non-significant increase in median OS (4, 5 months) and median PFS (2, 8 months) for Galinpepimut-S treated patients, as compared to the placebo arm (31). In July 2019, a clinical trial which investigates the combined treatment of Galinpepimut-S with nivolumab in patients with WT-1 expressing MPM (NCT04040231) has started.

## CRS-207

CRS-207 is a live-attenuated listeria-encoding human mesothelin (MSLN) vaccine. APCs will phagocytose the Listeria bacteria in CRS-207, leading to release of MSLN, that is subsequently presented by APCs to T-cells in the lymph nodes, thereby inducing an MSLN-specific immune response. MSLN is expressed in 90% of epithelioid MPM patients, which comprises up to 80% of all MPM patients (32). MSLN is not expressed in most sarcomatoid MPMs and only minimally in biphasic MPM. MSLN has low expression on non-malignant cells, making it an attractive target for immunotherapy (32, 33). In a phase Ib trial, treatment-naïve MPM patients received 2 CRS-207 doses, followed by 6 cycles of pemetrexed/cisplatin and CRS-207 booster infusions (34). The disease control rate was 89%, with 1 complete response (CR) and 19 partial responses (PR) in 35 evaluable patients. Unfortunate, the median OS was 14.7 months, which is comparable to OS observed after standard chemotherapy treatment (34, 35). Additional trials were initiated with CRS-207 in combination with pembrolizumab (Keytruda), chemotherapy and GM-CSF transfected tumor cell vaccine (GVAX) (NCT 01675765, NCT03175172, NCT02243371), and results are awaited (36). Unfortunately, the Keytruda trial has been halted because of insufficient clinical activity (NCT03175172).

In conclusion, despite careful selection of adjuvants and antigenic targets of cancer vaccines applied in MPM, therapeutic success or induction of a clinically detectable cytolytic immune response has not yet been shown (37). Combining cancer vaccines specifically with agents that target the immunosuppressive tumor microenvironment (TME) might improve clinical outcome. Clinical trials investigating these combination therapies are currently investigated and results are awaited.

## DC-therapy

DCs are low in numbers and are impaired in functionality in MPM patients (25). Moreover, the TME in MPM causes immunosuppression through secretion of immunosuppressive cytokines and expression of inhibitory molecules by tumor cells and immune cells again affecting DC mediated T-cell activation (38–41). To circumvent the immunosuppressive TME, DCs can be activated and loaded with selected tumor associated antigens (TAAs) or whole tumor lysate *in vitro*. DC-therapy has been developed in three generations. In first generation DC-therapy, monocytes isolated from peripheral blood were cultured with GM-CSF and interleukin (IL) 4, leading to the differentiation into immature monocyte-derived DCs (moDC) (42). These immature moDCs were loaded with TAAs or tumor lysate and reinjected without any further activating stimulation into the patient. Second-generation DC-therapy, additionally stimulated the generated moDCs *in vitro* with a maturation/activation cocktail, consisting of cytokines and immune stimulants, such as poly IC, TLR ligands and prostaglandin E2 (40–42). Second generation DC-therapy is currently used in various clinical trials. Response rates for second-generation DC-therapy in melanoma, prostate cancer, malignant glioma and renal cell carcinoma vary from 8 to 15% with an increase in OS of ~20% (42, 43). In contrary, an overall response rate of 7.1% was found in studies investigating first-generation DC-therapy in various malignancies, but mainly melanoma (44). Next-generation DC-therapy, aims at using naturally occurring DCs (nDC) that are purified directly from peripheral blood, *in vitro* loaded TAAs or tumor lysate and activated, and used for DC-therapy. The benefits of using nDCs are a shortened culture-time and lower manufacturing costs. It is also thought that DC-therapy containing nDCs will improve response rates, however this still has to be confirmed in clinical trials (42, 45, 46). DCs can be classically loaded with proteins during culture but TAAs can also be presented via RNA transfection methods or cancer cell-DC fusion (45, 47). The type of antigen source can vary from specific TAAs to complete tumor lysates. Analysis of 173 clinical trials in a wide variety of tumors showed that active immunotherapy using tumor-lysate (ORR 8.1%) was clinically more effective than peptide-based therapies (ORR 3.6%) (48), indicating that vaccinating with a broad range of tumor-associated proteins prohibits escape by the tumor and supports the hypothesis of immunoediting (Box 1).

Box 1 **Immunoediting.**

**Immunoediting** is a term that describes the balance between the prevention of tumor establishment through surveillance by the immune system and tumor cell growth when tumor cells escape from immunosurveillance (49–51).

Immunoediting by malignant cells contains three phases: elimination, equilibrium, and escape:

**Elimination:** cancer cells are eliminated by the innate and adaptive immune system.

**Equilibrium:** mutations and adaptations occur in certain cancer cells, leading to escape from the immune system of these cancer cells. During this phase, these mutated/adapted cancer cells will decrease antigen expression and become resistant to the immune system, whereas non-mutated cancer cells will be eliminated by the immune system, thereby increasing the frequency of mutated/adapted cancer cells. This process can take several years (52).

**Escape:** mutated/adapted cancer cells will proliferate and cause tumor outgrowth that can no longer be hampered or controlled by the immune system (53).

## DC-Therapy in MPM

Two types of second-generation DC-therapy have been tested in clinical trials in MPM patients. Autologous moDCs transfected with messenger RNA (mRNA) encoding for WT1 and autologous moDCs loaded with autologous/allogeneic tumor lysate.

### WT1-Targeted DC-Therapy

MoDCs transfected with WT1 encoding mRNA have resulted in promising clinical responses in MPM patients, but also in other malignancies. Prolonged stabilization of disease was noted in MPM patients, with OS (from start of chemotherapy) of 35.7 months (54, 55). This study was followed up by a phase I/II trial (MESODEC) in which treatment-naïve patients received WT1-targeting DC-therapy during chemotherapy, followed by pleurectomy/decortication (P/D) in the case of a resectable tumor (NCT02649829). The primary objective of this trial (recruiting since 2017 and enrolling 20 patients) is to assess the feasibility of WT1-targeting DC-therapy in combination with chemotherapy.

### Tumor Lysate Loaded DC-Therapy

Two clinical trials that applied DC-therapy that consists of autologous moDCs loaded with autologous tumor lysate have been reported in MPM (56, 57). In the first Phase I clinical trial, ten MPM patients were treated with at least 3 biweekly DC vaccinations. Tumor lysate was prepared from single cell suspensions of tumor cell lines generated from tumor tissue and/or pleural effusions. Three patients had a PR, one had stable disease (SD) and six had progressive disease (PD). Median OS from time of diagnosis was 19 months (57). To improve the efficacy of DC-therapy in a sequential trial, ten MPM patients were treated with a combination of moDCs loaded with autologous tumor lysate and low-dose cyclophosphamide treatment, a chemotherapy that at low concentration specifically targets regulatory T-cells (Tregs) that favor anti-tumor immune responses (40, 58–60). At first radiological evaluation after treatment, one patient had a CR, four had SD and two had PD. Radiological response assessment was impossible in three patients as they had received additional P/D (56). Grade III/IV toxicities did not occur. Moreover, cyclophosphamide treatment indeed selectively depleted Tregs and the frequency of naïve Tregs prior to treatment was positively correlated to OS (61). Two patients were still alive 6 years after diagnosis.

Unfortunately, using autologous tumor material as a source for tumor lysate is not feasible for a large number of patients in a phase II trial, because of the varying quality and/or lack of tumor material. Loading moDCs with allogeneic tumor lysate, serving as an “of-the-shelf” source for antigen-loading material, was compared to autologous tumor lysate-loaded moDC-therapy in mice, and induced similar protection against tumor outgrowth (62). To create allogeneic tumor lysate for clinical trials, cell lines were generated of pleural fluid of 5 MPM patients with different histological subtypes and varying antigen expression. An allogeneic tumor lysate was derived from these cell lines that contained a broad spectrum of TAAs. Two out of nine MPM patients treated with allogeneic tumor lysate-loaded moDCs (MesoPher) in a phase I dose-escalation trial had a PR and two patients are still alive 4 years after start of treatment. Grade III/IV toxicities were not reported (63). This phase I clinical study is followed up by an international, randomized, open-label, multicenter phase III trial (DENIM-trial), that will evaluate the efficacy of autologous moDCs loaded with allogeneic tumor lysate in MPM patients. Recruitment started in June 2018 and the first results are expected in 2021 (64). An overview of finished and ongoing clinical trials investigating DC-therapy in MPM is provided in Table 1.

**Table 1:** Ongoing and completed trials for dendritic cell therapy in mesothelioma.

**NCT nr**.	**Study type**	**Antigen**	**Type of DC**	**Additional therapy**	**Current status**	**Delivery method**	**Cancer type**	***n***	**Outcome**	**References**
NCT00280982	Phase 1	Autologous tumor lysate	Autologous moDC	None	Completed	i.v./i.d.	MPM	10	Pos tAE: moderate fever, no grade III/IV tox 3PR, 1SD, 6PD	(57)
NCT01241682	Phase 1	Autologous tumor lysate	Autologous moDC	Cyclo-phosphamide	Completed	i.v./i.d.	MPM	10	Pos tAE: moderate fever, no grade III/IV tox 7/10 patients with an OS ≥ 24 months	(56)
NCT02395679	Phase 1	Allogeneic tumor lysate	Autologous moDC	None	Completed	i.v./i.d.	MPM	9	Pos tAE: moderate fever, no grade III/IV tox Median PFS 8.8 months, 2PR, 7SD	(62)
NCT01291420	Phase 1	WT-1	autologous moDC	None	Completed	i.d.	MPM	10	Pos tAE; mild skin reactions, no grade III/IV tox 18-month survival rate: 75%	(54)
NCT03610360	Phase 3	Allogeneic tumor lysate	AUTOLOGOUS MODC	NONE	Recruiting	i.v./i.d.	MPM	230	-	
NCT02649829	Phase 1/2	WT-1	Autologous DC	First-line chemotherapy optional P/D	Recruiting	i.d.	MPM	20	-	
NCT03546426	Phase 1b	Autologous tumor homogenate	Autologous moDC	Pembrolizu-mab, IL-2	Not yet recruiting	i.d.	PD-L1 negative MPM	18	-	

*WT-1, Wilms Tumor 1; MPM, malignant pleural mesothelioma; n, expected number of patients; tAE, treatment related adverse event; i.d., intradermal; i.v., intravenous; pos, positive; BOR, best overall response; PD, progressive disease; tox, toxicity; SD, stable disease; CR, complete response; PR, partial response; OS, overall survival; PFS, progression-free survival; P/D, pleurectomy/decortication; moDC; monocyte-derived dendritic cell; DC, dendritic cell*.

### Combination Treatment DC-Therapy

Multiple reviews have discussed strategies to combine DC-therapy with other therapeutic agents, such as low-dose chemotherapy to deplete specific immune cell subsets, radiotherapy to induce an abscopal effect or therapies that target specific immune cell subtypes or enzymes (40, 41). CI-treatment is thought to not only complement DC-therapy but work synergistically with DC-therapy. Mice treated with DC-therapy had more tumor-specific CD8^+^ TILs than mice treated with placebo (65). Moreover, most of these TILs expressed high levels of PD1 on the cell surface, indicating their susceptibility for reinvigoration by CI treatment (65). The increase of TILs induced by DC-therapy may improve the current response rates of CI-treatment in MPM. Moreover, TILs induced by DC-therapy, that are hampered by inhibitory signaling may be reinvigorated. Based on this rationale, nine MPM patients who received autologous DC therapy in our center were sequentially treated with CIs. Three patients had a PR, five had SD and the median OS was 17.5 months from start of CI treatment (66). This data suggests a synergistic effect between DC-therapy and CIs in MPM that warrants further research.

## CAR T-cell Therapy

The hypothesis for adoptive T-cell therapy is to introduce tumor-specific T-cells that directly target the tumor cells. The first step toward CAR T-cell therapy was the use of autologous TILs that were expanded *in vitro* and reinjected after one dose of cyclophosphamide and in combination with IL-2 to treat metastatic melanoma. Objective regression was observed in 11 out of 20 patients with a mean response duration of 5.6 months (2–13 months) (67). Unfortunately, the reproducibility and quality of these TILs could not be guaranteed due to interpatient differences of TILs (68). To avoid the need of TILs, T-cells can be genetically modified to express a T-cell receptor (TCR) that targets tumor-specific antigens. Although promising radiological responses were observed using these transgenic TCR T-cells, clinical use was still restricted to (Human Leukocyte Antigen A2) HLA-A2 patients (69). In an effort to enhance the efficacy of transgenic TCR T-cells and make target-antigen recognition independent of (Major Histocompatibility Complex) MHC, a CAR instead of a TCR was developed (70). A CAR classically consists of an extracellular part with an antigen-recognition domain, a transmembrane domain and an intracellular domain that contains three immune receptor tyrosine-based activation motifs (ITAMs). CAR constructs are transfected into (autologous) T-cells via mRNA or viral transduction (71). Historically, five generations of CAR T-cell therapy are distinguished. The most crucial adjustments that separate different generations concern the characteristics of the intracellular domain, which can contain, apart from the three ITAMs, one or two co-stimulatory molecules, such as CD28 or 4-1BB, and an inducible expression cassette for a protein, as IL-12 or a cytokine receptor, such as IL-2R (72, 73). Currently, two second generation CAR T-cell therapies targeting CD19 have been approved for the treatment of hematological malignancies (74). Although the clinical outcomes for CAR T-cell therapy in treatment-resistant hematological malignancies are impressive with complete response rates varying from 40 to 60%, these responses are not found for solid tumors. Also, CAR T-cell therapy induces severe treatment-related toxicities varying from 49 to 73% (75–77). Cytokine release syndrome (CRS) and neurological events are the most frequent severe treatment-related adverse events. CRS results from an immense release of cytokines from immunotherapy-targeted immune cells and cancer cells. The severity of CRS is dependent on the dosage of CAR T-cells, amount of tumor burden and level of IL-6. Blocking the IL-6 receptor with tocilizumab or neutralizing IL-6 through binding with a mAb siltuximab reduces CRS severity (74). The mechanism driving neurotoxicity, CAR T-cell Related Encephalopathy Syndrome (CRES), is still unknown. Locoregional admission of CAR T-cell therapy could reduce toxicity, however for hematological malignancies this is not an option.

### Challenges for CAR T-Cell Therapy in Solid Tumors

CAR T-cell therapy encounters many challenges in solid tumors, such as migration of the CAR T-cells to the tumor, infiltration into the tumor, survival within the immunosuppressive TME as well as the lack of specific targetable tumor-specific antigens (78, 79). In B-cell driven malignancies, CD19 is a perfect target because it is expressed on all tumor cells (80, 81). Finding the perfect tumor-specific antigen to target in solid tumors is challenging due to heterogeneous expression of these tumor antigens. The lack of specific tumor antigens can also lead to severe “on target, off tumor” toxicity, caused by destruction of non-malignant cells expressing the antigen CAR T-cells are directed against (79). To migrate to and infiltrate the TME, CAR T-cells need to be equipped with appropriate tumor homing chemokine receptors and tumor endothelium degrading enzymes. Additionally, chemokines can be injected into the tumor that attract CAR T-cells. Another possibility to circumvent migration difficulties and even avoid development of systemic toxicities is locoregional administration of CAR T-cell therapy, but this is technically not achievable for all solid tumors. The stromal cells that are associated with nearly all epithelioid solid tumors form a physical barrier and severely hamper immune cell infiltration (79). A promising approach to attack the stromal component of the TME, is the development of CAR T-cells targeting (fibroblast activation protein) FAP which is expressed on various stromal cell types (82). Targeting the stromal cells by the FAP-specific CAR T-cells will allow and lead to infiltration of the tumor by TILs. Furthermore, as the target is expressed on non-malignant cells and not the malignant cells, this also reduces the risk of immunoediting and tumor escape. The immunosuppressive environment generated by the TME also affects the cytolytic activity of CAR T-cells and leads to CAR T-cell exhaustion. Secretion of inflammatory cytokines by CAR T-cells could counteract this immunosuppressive environment. Another possibility to directly circumvent exhaustion is to combine CAR T-cell therapy with CI treatment. Recently, CAR T-cells have been genetically modified with silenced PD-(L)1 coinhibitory signaling by the expression of a dominant negative PD1 receptor (DNPD-1R) that lacks an intracellular signaling domain. Although many challenges remain in the treatment of solid tumors with CAR T-cell therapy, current understanding and recent developments show great potential. Many of these new approaches are currently investigated in MPM.

### Systemic and Locoregional CAR-T Cell Therapy in MPM

The choice of targetable tumor-antigen is crucial in the development of CAR-T cell therapy for MPM. Several tumor-antigen targets, such as MSLN, WT-1, FAP and the antigens of the ErbB family are evaluated for their applicability for CAR T-cell therapy in MPM. CAR T-cells targeting MSLN, FAP or WT-1 are already investigated in clinical trials, summarized in Table 2. Second generation CD28 FAP CAR T-cells have been evaluated in a phase I trial. Patients with metastatic MPM treated with these CAR T-cells developed no treatment related toxicities. Radiological responses were not reported, but 2 out of 3 patients were still alive with a median follow up of 18 months. Recently, Haas et al. showed that treatment with second generation, 4-1BB MSLN CAR T-cells as monotherapy or in combination with low-dose cyclophosphamide was well-tolerated in patients with MPM, ovarian carcinoma and pancreatic ductal carcinoma (84). One case of dose limiting toxicity (grade 4 sepsis) was reported without the use of cyclophosphamide. No radiological responses were seen and 11 out of 15 patients had SD as best overall response. Moreover, the persistence of CAR T-cells in the peripheral blood was <28 days after injection. Apart from the known hurdles for CAR T-cell therapy in solid tumors, a potential reason for the minimal persistence and clinical efficacy might be a consequence of the murine-derived CAR that was used. A new phase 1 trial has started evaluating a fully human CAR T-cell (Table 1, NCT03054298). CAR T-cells targeting the ErbB family antigens, T1E28z CAR T-cells showed promising results both *in vitro* and in mouse models, which needs to be validated in a clinical studies (86–88).

**Table 2:** Ongoing and completed trials for T-cell therapy in mesothelioma.

**NCT nr**.	**Study type**	**Antigen**	**Stimulatory signal**	**Additional therapy**	**Current status**	**Delivery method**	**Cancer type**	***n***	**Outcome**	**References**
NCT01722149	Phase 1	FAP	CD28	Neoadjuvant chemotherapy	Completed, no results posted	i.p.	MPM	3*	Pos: tAE: none	(82)
NCT01355965	Phase 1	MSLN	4-1BB	ns	Completed, no results posted	i.v.	MPM, pancreatic cancer	18*	Pos: (only reported outcomes of 2 patients): tAE: none	(83)
NCT01583686	Phase 1/2	MSLN	ns	Fludarabine, cyclophosphamide, aldesleukin	Terminated	i.v.	MSLN expressing tumors	15*	Terminated due to slow accrual (14/15 patients had a BOR of PD)	-
NCT02159716	Phase 1	MSLN	4-1BB	Cyclophosphamide	Completed, no results posted	i.v.	MPM, pancreatic cancer and ovarian cancer	15*	Pos: tAE: 1 grade IV tox 11SD, 4PD	(84)
NCT02580747	Phase 1	MSLN	ns	ns	Unknown	ns	MSLN expressing tumors	20	-	
NCT02930993	Phase 1	MSLN	ns	Cyclophosphamide	Unknown	i.v.	MSLN expressing tumors	20	-	
NCT03638206	Phase 1	MSLN	ns	Fludarabine, cyclophosphamide	Recruiting	ns	MPM	ns	-	
NCT03054298	Phase 1	MSLN	ns	cyclophosphamide	Recruiting	i.v./i.p.	MSLN expressing tumors	30	-	
NCT02408016	Phase 1	WT-1	ns	Cyclophosphamide, surgery IL-2	Active, not recruiting	i.v.	MPM/NSCLC	20	-	
NCT03615313	Phase 1/2	MSLN	PD-1 excreting CAR T cells	Fludarabine, cyclophosphamide	Recruiting	i.v.	MSLN expressing tumors	50	-	
NCT02414269	Phase 1/2	MSLN	CD28	Cyclophosphamide, pembrolizumab	Recruiting	i.p.	MPM	179 21***	Pos: tAE: no grade III/IV tox 2 CR, 5 PR, 4 SD, 10 PD	(85)
NCT03907852	Phase 1/2	MSLN	TRuC (novel T cell engenering platform)	Cyclophosphamide, pembrolizumab, fudarabine	Recruiting	ns	MSLN expressing tumors	70	-	
NCT03925893	Phase 2	-	TIL	Fludarabine, cyclophosphamide, aldesleukin	Recruiting	i.v.	Solid tumors	10	-	
NCT02414945	Phase 1/2	-	TIL	Fludarabine, cyclophosphmide, IL-2	Recruiting	i.v.	MPM	10	-	

FAP, fibroblast activation protein; MSLN, mesothelin; WT-1, Wilms Tumor 1; MPM, malignant pleural mesothelioma; n, expected number of patients; *actual enrollment; ***21 patients were enrolled in the phase I trial which was reported at the AACR 2019; TRuC, T Cell Receptor Fusion Constructs; ns, not specified; TILs, tumor infiltrating lymphocytes; tAE, treatment related adverse event; i.p., intrapleural; i.v., intravenous; pos, positive; BOR, best overall response; PD, progressive disease; tox, toxicity; SD, stable disease; CR, complete response; PR, partial response.

Currently methods to improve migration to the tumor site are heavily studied in mouse models. Herein, MSLN CAR T-cells that expressed a tumor homing chemokine receptor CCR2 showed improved tumor infiltration (89). Moreover, in an orthotopic mouse model of MPM, migration toward the tumor was circumvented by intra-pleural administration of second generation, CD28-costimulated MSLN CAR T-cells and led to a larger reduction of pleural an metastatic tumor load as compared to intravenous administration (90). Moreover, the intra-pleural treatment dose was 30-fold lower than the intravenous administered dose and elicited no grade III/IV toxicities.

In a clinical setting, no'on-target, off-tumor' effects were seen when 21 patients with malignant pleural disease were treated with CD28-costimulated MSLN CAR-T cells intrapleurally (85, 90, 91). In this study 19 out of 21 patients had MPM, of whom 13 were subsequently treated with pembrolizumab (anti-PD1). In total two patients had a CR, five had PR and four had SD as best overall response (85). Just as for DC therapy, Combining CAR T-cell therapy with anti-PD1 treatment showed promising clinical results. In a MPM mouse model, combined treatment of anti-PD1 mAb with CAR T-cell therapy improved treatment efficacy. CAR T-cell exhaustion can also be prevented by genetically modifying the CAR T-cells to express a dominant negative PD1 receptor (DNPD-1R) that lacks an intracellular signaling domain, avoiding the need for CI treatment and their related toxicities (92). A trial with CAR T-cells with a DNPD1R is expected to start in 2020 (93).

## Conclusions

MPM remains a treatment-recalcitrant tumor with few registered treatment options. CI treatment failed to improve clinical outcome which might correlate with the low number of TILs in MPM. Cancer vaccines, DC-therapy and CAR T-cell therapy all induce a tumor directed immune response and increase the number of tumor-specific T-cells. Both cellular therapies and cancer vaccines face many challenges such as, migration of therapy-induced T-cells to the tumor, infiltration into the tumor, survival within the immunosuppressive TME and finding an optimal targeting approach. Improvement of cancer vaccines and cellular therapies and multimodal approaches that circumvent and overcome these difficulties should be investigated thoroughly. As both cancer vaccines and cellular therapies aim to induce infiltration of tumor-specific T cells into the TME, CI treatment serves as an ideal therapeutic option to block inhibitory signaling and reinvigorate TILs leading to enhancement of both treatments. In conclusion, additional research is needed to investigate and compare effectivity of cancer vaccines and cellular therapies for a cold tumor like MPM. Evaluating and influencing characteristics of the TME in MPM that withhold T-cell infiltration or impair cytotoxic T-cell function, is warranted to create a holistic treatment approach.

## Author Contributions

RB drafted and wrote the paper and contributed to the conception of the work. JA and HV contributed to the conception of the work and substantively revised the manuscript. All authors approved the submitted version.

## Conflict of Interest

JA reports receiving commercial research grants from Amphera and Roche, holds ownership interest (including patents) in Amphera BV, and is a consultant/advisory board member for Amphera, Boehringer Ingelheim, Bristol-Myers Squibb, Eli-Lilly, MSD, and Roche. The remaining authors declare that the research was conducted in the absence of any commercial or financial relationships that could be construed as a potential conflict of interest.
